# Transcriptome analysis of the Holly mangrove *Acanthus ilicifolius* and its terrestrial relative, *Acanthus leucostachyus,* provides insights into adaptation to intertidal zones

**DOI:** 10.1186/s12864-015-1813-9

**Published:** 2015-08-14

**Authors:** Yuchen Yang, Shuhuan Yang, Jianfang Li, Yunfei Deng, Zhang Zhang, Shaohua Xu, Wuxia Guo, Cairong Zhong, Renchao Zhou, Suhua Shi

**Affiliations:** State Key Laboratory of Biocontrol and Guangdong Provincial Key Laboratory of Plant Resources, Sun Yat-sen University, Guangzhou, 510275 China; Key Laboratory of Plant Resource Conservation and Sustainable Utilization, South China Botanical Garden, Chinese Academy of Science, Guangzhou, 510650 China; Hainan Dongzhai Harbor National Nature Reserve, Haikou, 571129 China

**Keywords:** Abiotic stress, Adaptation, Mangroves, Comparative transcriptome

## Abstract

**Background:**

*Acanthus* is a unique genus consisting of both true mangrove and terrestrial species; thus, it represents an ideal system for studying the origin and adaptive evolution of mangrove plants to intertidal environments. However, little is known regarding the two respects of mangrove species in *Acanthus*. In this study, we sequenced the transcriptomes of the pooled roots and leaves tissues for a mangrove species, *Acanthus ilicifolius*, and its terrestrial congener, *A. leucostachyus*, to illustrate the origin of the mangrove species in this genus and their adaptive evolution to harsh habitats.

**Results:**

We obtained 73,039 and 69,580 contigs with N50 values of 741 and 1557 bp for *A. ilicifolius* and *A. leucostachyus*, respectively. Phylogenetic analyses based on four nuclear segments and three chloroplast fragments revealed that mangroves and terrestrial species in *Acanthus* fell into different clades, indicating a single origin of the mangrove species in *Acanthus*. Based on 6634 orthologs, *A. ilicifolius* and *A. leucostachyus* were found to be highly divergent, with a peak of synonymous substitution rate (Ks) distribution of 0.145 and an estimated divergence time of approximately 16.8 million years ago (MYA). The transgression in the Early to Middle Miocene may be the major reason for the entry of the mangrove lineage of *Acanthus* into intertidal environments. Gene ontology (GO) classifications of the full transcriptomes did not show any apparent differences between *A. ilicifolius* and *A. leucostachyus*, suggesting the absence of gene components specific to the mangrove transcriptomes. A total of 99 genes in *A. ilicifolius* were identified with signals of positive selection. Twenty-three of the 99 positively selected genes (PSGs) were found to be involved in salt, heat and ultraviolet stress tolerance, seed germination and embryo development under periodic inundation. These stress-tolerance related PSGs may be crucial for the adaptation of the mangrove species in this genus to stressful marine environments and may contribute to speciation in *Acanthus*.

**Conclusions:**

We characterized the transcriptomes of one mangrove species of *Acanthus*, *A. ilicifolius*, and its terrestrial relative, *A. leucostachyus*, and provided insights into the origin of the mangrove *Acanthus* species and their adaptive evolution to abiotic stresses in intertidal environments.

**Electronic supplementary material:**

The online version of this article (doi:10.1186/s12864-015-1813-9) contains supplementary material, which is available to authorized users.

## Background

Tropical intertidal zones are extreme environments characterized by high salinity, drought, hypoxia and high ultraviolet (UV) radiation, which severely limit plant growth, development and reproduction [1]. As the dominant forest community and ecosystem in the coasts, mangrove plants struggle and survive in these environments with remarkable morphological and physiological characteristic, for example, exposed breathing roots, support roots and buttresses, salt-excreting leaves, and viviparous water-dispersed propagules [1, 2]. Understanding the genetic basis underlying those adaptive traits at the genomic level could provide important clues to the molecular mechanisms of stress resistance in marine halophytes. Mangroves are constituent plants of approximately 70 species from 28 genera belonging to 20 families [3]. Studies based on fossils and phylogenetic analysis have suggested that these biogeographically and taxonomically diverse genera are of independent origins in different geologic epochs [4, 5]. However, the divergent time and the species radiation within some genera are still unclear and are of great interest to many botanists.

Comparative analysis among mangrove species and model terrestrial plants using RNA-sequencing has revealed that specific sequence divergence and transcriptional regulation play major roles in response to salt stress in many mangrove species, such as *Aegiceras corniculatum* [6], *Bruguiera gymnorhiza* [7, 8] and *Sonneratia alba* [9]. However, the model plants used in these studies, such as *Arabidopsis thaliana* and *Populus tomentosa*, are distantly related to mangroves, which makes it difficult to distinguish adaptive processes from those caused by phylogenetic effects, thus reducing the resolution of selection signals [10].

Among all true mangrove genera, *Acanthus* is the only genus that includes both mangrove and terrestrial species; therefore, it is an ideal system to investigate the adaptive evolution of mangrove plants to stressful intertidal environments while minimizing phylogenetic influences. This genus consists of three representative mangrove species and approximately 27 terrestrial species [1]. The three mangrove species, which are also called Holly mangroves, are found in the intertidal zones from India to the West Pacific and tropical Australia. Unlike other terrestrial species of *Acanthus*, which are distributed in the Mediterranean Basin, *A. leucostachyus* is restrict to South to Southeast Asia and grows under rain forest canopies at the edges of streams 600–1200 m above the sea level [11]. Similarities in geographic distribution and ecological requirements of *A. leucostachyus* and the congeneric mangrove species suggested *A. leucostachyus* is a suitable outgroup for illustrating the evolutionary history of the mangrove species in *Acanthus*.

Nguyen et al. [12, 13] identified 170 genes that are involved in response to salt stress from 628 expressed sequence tags (ESTs) of *A. ebracteatus*. However, these studies only listed the annotations of these stress-response genes. The role of natural selection in adaptive evolution of mangrove species has not been characterized. In the current study, we performed RNA-sequencing to assess the mangrove species *A. ilicifolius* and its terrestrial congener *A. leucostachyus*, to provide insights into the evolutionary process underlying the adaptation of mangroves to intertidal zones. We asked the following specific questions: 1) what is the origination pattern of the mangrove *Acanthus* taxa and when mangrove species diverged from their terrestrial relatives within this genus? 2) Are there any marked differences in Gene Ontology (GO) classification among the mangrove transcriptome profiles? Finally, 3) which genes have experienced adaptive evolution and contributed to the adaptation of *A. ilicifolius* to stressful intertidal habitats?

## Results

### Transcriptome sequencing and de novo assembly

A total of 44.04 million 90-bp and 46.89 million 100-bp paired-end reads were sequenced for *A. ilicifolius* and *A. leucostachyus*, respectively (Table 1). The raw data were deposited in the NCBI Sequence Read Archive (SRA) with the accession number SRP053334. To minimize sequencing errors, we individually trimmed each read to its longest contiguous segment, using quality scores of higher than 20 for all remaining segments. After the trimming processes, reads with length of less than 50 bp were removed from each dataset. A total of 34.13 and 38.53 million high-quality reads for *A. ilicifolius* and *A. leucostachyus*, respectively, were used for a further *de novo* assembly. This assembly was performed with Trinity software [14], and generated 97,347 and 90,998 contigs for the two species, respectively. Of them, 24,487 and 19,212 similar contigs were clustered into 4869 and 2633 clusters by TGICL [15] using a default threshold of 0.94 and CD-HIT [16] using a global identity threshold of 0.90. A total of 77,729 and 74,419 contigs were remained in the two datasets, respectively.

**Table 1:** Sequencing and assembly statistics for the transcriptome data of *Acanthus ilicifolius* and *A. leucostachyus*. No. is short for number

	Assembly results		After removing redundancy and contigs with low coverage or depth	
	*A. ilicifolius*	*A. leucostachyus*	*A. ilicifolius*	*A. leucostachyus*
No. of contigs	97347	90998	73039	69580
Maximum length of contigs (bp)	5243	11541	5243	11541
Average length of contigs (bp)	601	980	580	913
Contig N50 (bp)	816	1699	763	1580
No. of contigs with length greater than 1 kb	15755	31572	10655	21776

To enhance the reliabilities of the assembly results, we calculated the coverage and the average depth for each contig and removed 4690 and 4839 contigs with less than 75 % sites with at least 2× depth from the two data sets, respectively. At last, 73,039 and 69,580 unigenes with average depths of 32× and 31× were retained for *A. ilicifolius* and *A. leucostachyus*, respectively. The average lengths of the remained contigs were 580 and 913 bp and N50 values were 763 and 1580 bp, respectively, suggesting that the transcriptomes for both species were of good quality. The discrepancy in N50 values between the two species may be due to the difference of the genomic complexity. The length distribution of these contigs is shown in Additional file 1.

### Phylogenetic analysis and the divergent time estimation

**Fig. 1:**
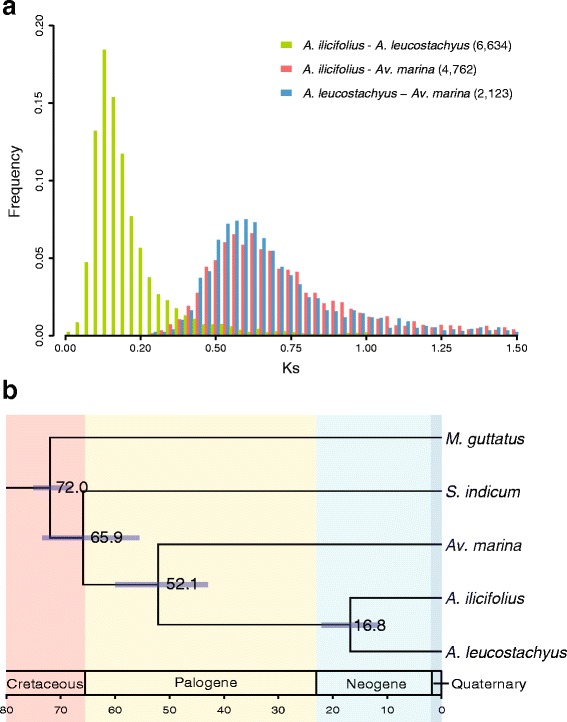
Genetic divergence and the divergent time among *Acanthus ilicifolius*, *A. leucostachyus* and *Avicennia marina*. **a** Synonymous substitution rates (Ks) distribution of the orthologs between each pair of *Acanthus ilicifolius*, *A. leucostachyus* and *Av. marina*. The numbers in parentheses after the species name indicate the number of orthologs used for Ks distribution plotting. **b** Divergent time of *A. ilicifolius*, *A. leucostachyus* and *Av. marina*, as well as the two outgroups *Sesamum indicum* and *Mimulus guttatus*. The scale bar is 10 million years. The value and purple bar at each node indicate the estimated divergent time (million years) with a 95 % credibility interval

In this study, we estimated the synonymous substitution rate (Ks) of the orthologs between each pair of the two *Acanthus* species and *Avicennia marina* (*Av. marina*). The results showed that the peak of Ks distribution was 0.145 between *A. ilicifolius* and *A. leucostachyus*, while it was 0.605 and 0.585 between *Av. marina* and *A. ilicifolius* and *A. leucostachyus*, respectively (Fig. 1a). This unusual high Ks value between *A. ilicifolius* and its terrestrial congener suggests long-term divergence, albeit within the same genus. Our estimation indicated that *A. ilicifolius* diverged with *A. leucostachyus* at approximately 16.8 million years ago (MYA) with a 95 % credibility interval (CI) of 11.6 to 22.1 MY, while the split time of *Acanthus* and *Avicennia* was dated at 52.1 MYA (95 % CI: 43.0–60.0). The estimated divergence time between Acanthaceae s.l. and Pedaliaceae was approximately 65.9 MYA (95 % CI: 55.6–73.4).

**Fig. 2:**
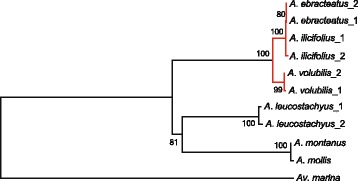
Phylogenetic tree of six species of *Acanthus*. The branches of mangroves species were marked in red. Numbers at each node indicate maximum likelihood bootstrap values

To infer the origin of the mangrove species in *Acanthus*, we reconstructed the phylogenetic tree for the three mangrove species, *A. ilicifolius*, *A. ebracteatus* and *A. volubilis*, and their terrestrial relatives, *A. leucostachyus* and two European species *A. mollis* and *A. montanus*, based on the combined sequences of four nuclear segments, including three nuclear genes (CL302, CL4763 and c51040) and the nrITS region, and the three chloroplast fragments (*trn*V-*trn*M, *rpl*-*rps* and *trn*L-*trn*F). Maximum likelihood analysis revealed that *Acanthus* fell into two clades with high bootstrap support (BS); three mangrove species formed one clade, and the three terrestrial species formed the other (BS = 100 and 81 %, respectively; Fig. 2). Within the mangrove clade, *A. ebracteatus* formed a single clades with a BS value of 80 %. Compared to *A. volubilis*, *A. ilicifolius* and *A. ebracteatus* was close to each other with strong support (BS = 100 %). Within the terrestrial clade, *A. mollis* is sister to *A. montanus* with 100 % BS.

Furthermore, we calculated the Ks for the combined sequences of the three nuclear genes and the Kimura two-parameter distances for the nrITS and chloroplast fragments (Additional files 2, 3 and 4). For the three nuclear genes, the average Ks between *A. ilicifolius* and *A. leucostachyus* was 0.035. Based on the divergence time of the two species of 16.8 MYA, the synonymous substitution rate within Acanthus was estimated to be 1.04e-9 per synonymous site per year. According to this synonymous substitution rate, the splitting time between the mangrove clade and the two European terrestrial species was estimated at 35.1 MYA. Within the mangrove clade, the divergence time between *A. ilicifolius* and *A. ebracteatus* (Ks = 0.0045) was estimated to be 2.2 MYA, while it was estimated to be 2.3 MYA between *A. volubilis* and the clade of *A. ilicifolius* and *A. ebracteatus* (Ks = 0.00475). Comparatively, the divergence of *A. leucostachyus* and the clade of *A. montanus* and *A. mollis* was 0.049, corresponding to 23.6 MYA. Similarly, for nrITS and the combined chloroplast fragments, the mean of the Kimura two-parameter distances between the mangrove and terrestrial clades were 0.096 and 0.029, which are one order of magnitude higher than those among the three mangrove taxa (0.012 and 0.001), respectively.

### Functional annotation

**Fig. 3:**
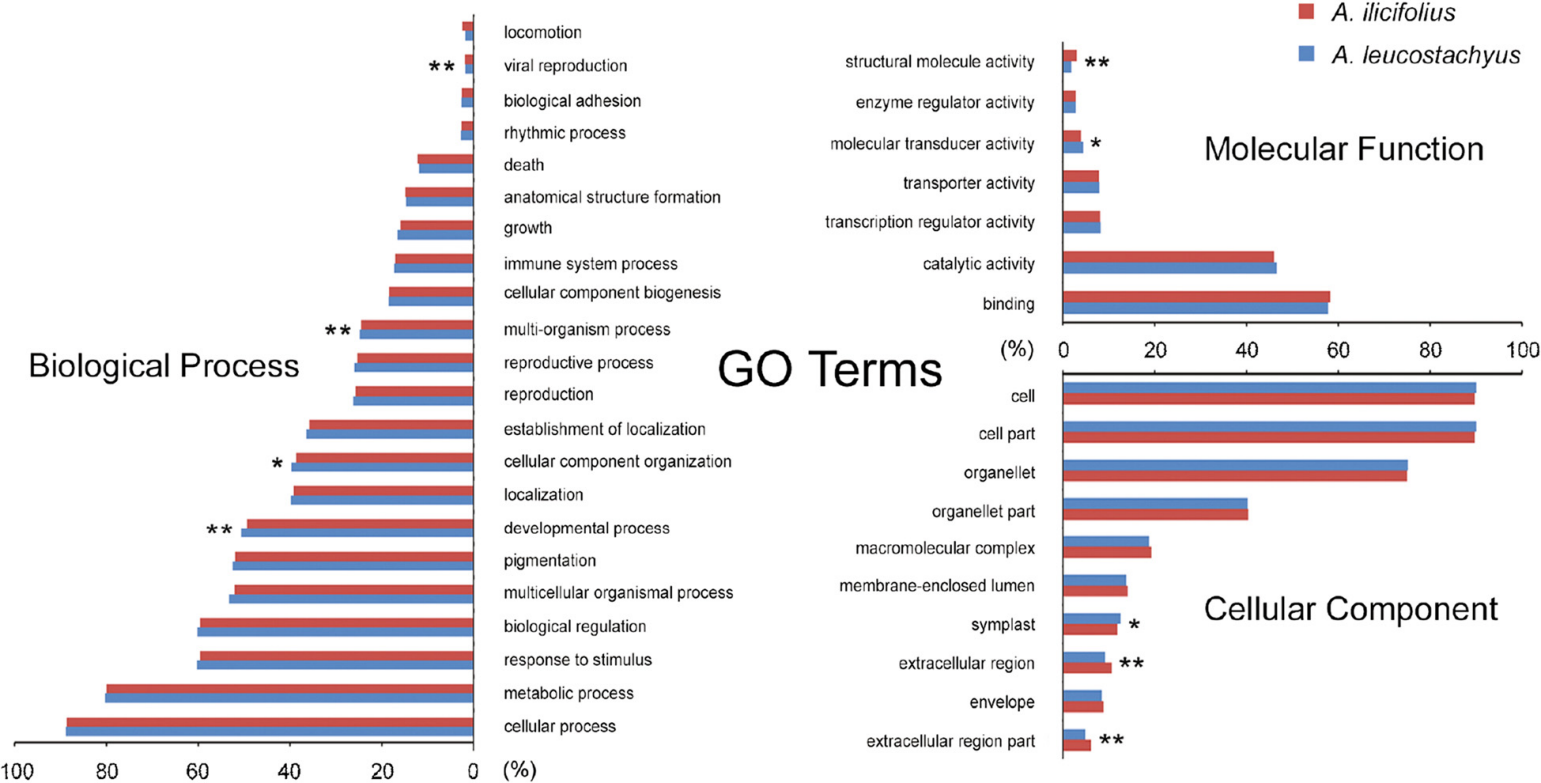
Gene Ontology (GO) distributions for *Acanthus ilicifolius* (red) and *A. leucostachyus* (blue). Annotation results were mapped to categories in the second level of GO terms, respectively. GO terms that contain less than 1 % of total genes were excluded from this graph. *, p-value <0.05. **, p-value < 0.01

To access the GO classification for each gene, the full transcriptome sequences of *A. ilicifolius* and *A. leucostachyus* were annotated with the Swiss-Prot database in AgBase [17] with a cutoff e-value of 1e-6. A total of 29,244 (40.04 %) and 25,864 (37.2 %) genes, respectively, were successfully annotated with known GO terms. The GO distributions for the two species are shown in Fig. 3. In general, the GO classifications did not show significant differences between the transcriptome profiles of *A. ilicifolius* and *A. leucostachyus*. In the cellular component category, cell and organelle component-related functions were predominant. A total of 26,335 (90.1 %) and 23,206 (89.7 %) genes were assigned to cell and 26,335 (90.1 %) and 23,206 (89.7 %) to cell part for the two species, respectively. There were 21,979 and 11,797 genes in *A. ilicifolius* and 19,394 and 10,450 genes in *A. leucostachyus* annotated with organelle and organelle part. In molecular function category, binding and catalytic activity were the most enriched, comprising 16,896 (57.8 %) and 13,629 (46.6 %) genes in *A. ilicifolius* and 15,054 (58.2 %) and 11,904 (46.0 %) genes in *A. leucostachyus*, respectively. In the last category, biological process, 26,064 (89.1 %), 23,574 (80.6 %) and 17,676 (60.4 %) genes were annotated to three major GO terms in *A. ilicifolius*, including cellular and metabolic process and biological regulation, while 22,999 (88.9 %), 20,745 (80.2 %) and 15,475 (59.8 %) genes were assigned to these three terms in *A. leucostachyus*. It should be noted that 17,694 (60.5 %) and 15,461 (59.8 %) genes were assigned to the GO term response to stimulus, which also comprised a large proportion of the biological process category for both species. These results indicate that functions with enriched annotation may be quite fundamental and essential for plants.

Several GO terms significantly differed between *A. ilicifolius* and *A. leucostachyus*. Compared with *A. leucostachyus*, a lower percentage of genes in the cellular component category were annotated with extracellular region and extracellular region part (*p* < 0.01), and a higher percentage to symplast (*p* < 0.05) for *A. ilicifolius*. In the molecular function category, genes related to molecular transducer activity were more abundant in *A. ilicifolius* compared with *A. leucostachyus* (*p* < 0.05), but those involved in structural molecular activity were less prominent (*p* < 0.01). In the biological process category, more genes were assigned to multicellular organismal and development processes (*p* < 0.05) as well as cellular component organization (*p* < 0.01) in *A. ilicifolius* than in *A. leucostachyus*.

### Identification of genes under positive selection in *A. ilicifoliu*s

**Fig. 4:**
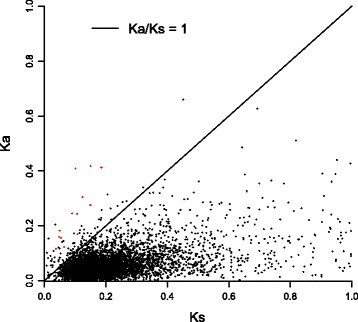
Ka/Ks distribution of 6634 pairs of orthologs between *Acanthus ilicifolius* and *Ac. leucostachyus*. The solid line marks Ka/Ks = 1, whereas the red dots mark the genes with Ka/Ks ratio significantly larger than 1

We calculated and plotted the non-synonymous to synonymous substitution ratio (Ka/Ks) for the 6634 pairs of orthologs between the two *Acanthus* species, *A. ilicifolius* and *A. leucostachyus*, as shown in Fig. 4. Only 13 pairs were identified with a Ka/Ks ratio that was significantly larger than 1, indicating signals of positive selection in these two species (red dots in Fig. 4). The functional descriptions of the 13 pairs are listed in Additional file 5. Of them, four were found to be involved in the response to biotic and/or abiotic stimuli (bold in Additional file 5).

**Fig. 5:**
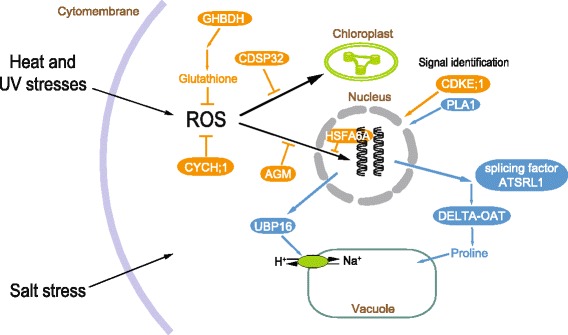
Positively selected and expanded key genes in stress resistance pathways of *Acanthus ilicifolius*. Genes, products and pathways for responding to salt-stress and for heat and ultraviolet-stress were marked in blue and orange shading, respectively

To identify candidate genes under positive selection along the branch of *A. ilicifolius*, we further tested the 2245 pairs of orthologs among four species, *A. ilicifolius*, *A. leucostachyus*, *Av. marina* and *S. indicum*, using the improved branch-site likelihood method in the codeml module of PAML [18–20]. A total of 99 genes were identified as positively selected genes (PSGs) in *A. ilicifolius*. GO annotations revealed that 48 (20.8 %) and 42 (18.2 %) of the 99 genes were assigned to metabolic and cellular process, respectively (Additional file 6a). Of the 99 genes, 18 sequences were assigned to 13 Kyoto Encyclopedia of Genes and Genomes (KEGG) pathways (15 unique EC numbers) [21]. Detailed annotations were performed on these genes and the results are listed in Additional file 7. Interestingly, 23 genes were annotated with functions involving stimulus responses and reproduction, and 16 were found to be directly involved in response to abiotic stresses (Fig. 5; bold in Additional file 7). These genes fell into three functional groups. The first group consisted of four genes involved in salt-stress resistance. One gene of interest, Ail_c57143_g1_i2, catalyzes the transfer of an amino group from ornithine to the precursor of proline, L-glutamate 5-semialdehyde, which plays a key role in the proline synthesis pathway. Twelve genes with annotations of response to heat and UV stress were assigned to the second groups. Two genes, Ail_c48553_g1_i1 and Ail_c56385_g1_i1, were found to be involved in the last step of the synthesis of glutathione, which is an important antioxidant that protects important cellular components from damage caused by reactive oxygen species (ROS) in plants. The third groups included seven genes involved in seed germination and embryo development.

We further calculated the fragments per kilobase of exon per million fragments mapped (FPKM) [22] for the 23 genes in *A. ilicifolius* and *A. leucostachyus* (Additional file 6b). We found that 18 of the 23 genes were expressed at low levels and that their expression levels did not differ between the two species. In contrast, three genes, Ail_c56385_g1_i1, Ail_c48553_g1_i1 and Ail_c52163_g1_i1, were expressed at significantly higher levels in *A. ilicifolius* than in *A. leucostachyus* (red double asterisks in Additional file 6b), and two gene, Ail_c53207_g1_i1 and Ail_c51326_g2_i1, showed decreased expression in *A. ilicifolius* than in *A. leucostachyus* (blue double asterisks in Additional file 6b).

## Discussion

### High divergence between the mangrove and terrestrial species within *Acanthus*

Most genera comprising mangrove species contain exclusively mangroves [3], and each genus is considered to be a monophyletic group. *Acanthus* is the only genus consisting of both true mangroves and terrestrial plants; thus, it is a good system for dissecting the adaptive evolution of mangroves. In this study, we reconstructed the phylogenetic tree for the genus *Acanthus*, including three mangrove species and three terrestrial relatives, and *Av. marina* was used as the outgroup. The topological structure revealed that the mangrove and terrestrial species of *Acanthus* fell into different clades, with the peak of Ks distribution of 0.145 (Fig. 1a). The pairwise Kimura two-parameter distances of the six *Acanthus* species also revealed that the divergence between the mangrove and terrestrial species of *Acanthus* is one order of magnitude higher than that among the three mangrove taxa (Additional files 3 and 4). The splitting time of *A. ilicifolius* and its terrestrial relative *A. leucostachyus* was estimated to be 16.8 MYA (Fig. 1b), while 35.1 MYA between the mangrove clade and the two European species. These estimations suggest that the mangrove *Acanthus* species entered the intertidal environment in Early to Middle Miocene (approximately 23.0 - 11.6 MYA). The transgression that occurred from 18.0 to 16.0 MYA caused dramatic environmental changes and the loss of tropical forests, which may be the major reason for new lineages entering the intertidal environment [23].

In contrast with the high divergence of the mangrove clade and their terrestrial relatives, the three mangrove Acanthus species had quite low interspecific divergence, especially between *A. ilicifolius* and *A. ebracteatus*. The average Ks between *A. volubilis*, and the clade of *A. ilicifolius* and *A. ebracteatus* was 0.00475, suggesting that the divergence time between these two clades was 2.3 MYA according to the assumed synonymous substitution rate of 1.04e-9 per synonymous site per year. The two sibling species, *A. ilicifolius* and *A. ebracteatus*, show very close but distinct relationship in the phylogenetic trees. This result is in agreement with the taxonomy based on morphological characteristics, including the open inflorescences, white or purple flowers, absent bracteoles and stem without axillary spines of *A. ebracteatus* [24], although these two species are considered to be the same species by some authors [25]. In this study, the divergence time between *A. ilicifolius* and *A. ebracteatus* was estimated as 2.2 MYA, based on the three nuclear genes. Such recent speciation has also been observed in several mangrove genera, such as *Sonneratia* [26]. However, unlike other mangrove species, *A. ilicifolius* and *A. ebracteatus* have nearly identical distributions, suggesting that the primary force driving the divergence of these two species cannot be simply attributed to geographic isolation but that there is a high probability of ecological or adaptive differentiation. However, this hypothesis requires a further testing in the future.

### Absence of genetic components specific to the mangrove transcriptomes

Resistance to extreme abiotic stress in the intertidal zone is a complex process involving numerous genes that underlie relevant the morphological and metabolic characteristics across the transcriptome. In the current study, we compared the GO classifications between the transcriptomes of *A. ilicifolius* and its terrestrial relative, *A. leucostachyus*. We did not observe any noteworthy differences in GO classifications between these two species, except for a small number of GO terms. Most of the genes were assigned to fundamental functions, such as cellular and organelle structure, binding and catalytic activity, metabolic process and biological regulation, in agreement with a previous report of the mangrove species *S. alba* [9]. However, these results are inconsistent with reports of two other mangrove species, *R. mangle* and *Heritiera littoralis*, which have been suggested to possess mangrove-specific genomic characteristics [27]. The discrepancy between our results and previous study may due to the species specificity and the differences in the tissue types for RNA sequencing. In *Acanthus*, we found that the GO classifications between the mangrove and terrestrial species were similar, suggesting few genetic components specific to the mangrove transcriptomes.

### Positively selected genes in *A. ilicifolius*

In the past decade, more and more studies have suggested that the differential adaptation of populations is the primary force driving population divergence and speciation [28–30]. Identifying and characterizing the pattern of selective pressures across a genome, such as sequence divergence, may provide insights into the processes and mechanisms of speciation [31–33]. In this study, although no genetic components specific to the mangrove transcriptomes were identified, we identified signals of natural selection in a few genes. According to the Ka/Ks ratios, 13 of the 6634 pairs of orthologs between two *Acanthus* species were found to be under positive selection (Additional file 5). Of them, one ortholog (Ail_c48842_g1_i2) was annotated as *TIP1;2*, a gene that is involved in response to salt stress. The expression of this gene in NaCl stress-sensitive yeast mutant strains has been shown to highly increase the resistance to NaCl treatment [34]. The ortholog Ail_c53470_g4_i1 was found to be a homolog of *GF14 omega*, which is considered to be involved in rescuing defects in DNA-damage and DNA-replication checkpoints and may play an important role in the oxidative stress response [35]. Another two orthologs, Ail_c48391_g1_i2 and Ail_c52685_g1_i1, function in response to attack by microbes [36, 37].

Furthermore, to investigate the genetic bases of the adaptive traits of *A. ilicifolius*, we employed the improved branch-site likelihood method and identified 99 candidate PSGs out of the 2245 orthologous genes in *A. ilicifolius*. In contrast with its terrestrial relative *A. leucostachyus*, *A. ilicifolius* is faced with three major abiotic stresses in the intertidal environment, high salinity, high temperatures and ultraviolet radiation, and periodic inundation. The annotations indicated that a total of 23 PSGs were involved in the responses to these three stress conditions (Fig. 5; bold in Additional file 7). Four genes that function in response to salt stress were assigned to the first group (blue shading in Fig. 5). Of them, Ail_c57143_g1_i2, annotated as ornithine aminotransferase, may play a key role in responding to salt stress in *A. ilicifolius*. The product of this gene transfers the amine group from ornithine to glutamate, a precursor of proline [38]. Proline is known to be the major osmolyte involved in response to salt stress in *A. ilicifolius*, as well as many other mangroves and terrestrial plants [39–41]. Another gene, Ail_CL2465Contig1, is homologous to the gene encoding ubiquitin-specific protease 16 (UBP16) of *Arabidopsis thaliana*, which could increase salt tolerance by positively regulating plasma membrane Na(+)/H(+) antiport activity under salt stress condition [42].

The second group consisted of 12 genes related to scavenging and the repairing of damage due to oxidative and superoxide stress and UV radiation under high temperature and light conditions (orange shading in Fig. 5). In the intertidal zone, high temperatures and ultraviolet radiation may result in the dramatic accumulation of ROS, leading to severe damage of DNA and cell structures [43]. Two of the ten genes, Ail_c48553_g1_i1 and Ail_c56385_g1_i1, are involved in the last step of the synthesis of glutathione, which is an important antioxidant in plants that protects vital cellular components from damage caused by ROS [44]. Another gene Ail_c51326_g2_i1 showed homology to the gene encoding the chloroplastic drought-induced stress protein of 32 kD (CDSP32) of *Solanum tuberosum*. CDSP32 is a thioredoxin that participates in defense against oxidative damage in photosynthetic membranes [45–47].

Seven genes involved in seed germination and embryo development were assigned into the third group. Zhang et al. [48] have revealed that short-term tide inundation of <15 h per day has no impact on the time of seed germination; however, long-term inundation of ≥15 h per day delays seed germination and decreases the germination rate in *A. ilicifolius*. Genes Ail_CL5647Contig1 is homologous to *BRIZ2* of Arabidopsis thaliana. *BRIZ2* is involved in forming a heteromeric E3 ligase complex, which is required for seed germination and post-germination growth [49]. Another three genes Ail_CL3016Contig1, Ail_CL3870Contig1 and Ail_c54714_g1_i1 were found to play important role in embryo development [50–52]. Thus, these genes may greatly improve the reproductive success of *A. ilicifolius* in harsh coastal environments. These three groups of resistance related PSGs are crucial to the survival and adaptation of *A. ilicifolius* to extreme habitats. Adaptation to the differential environments might be the primary force driving species diversification in *Acanthus*.

The FPKM values of the most of the 23 genes were low for both *A. ilicifolius* and *A. leucostachyus*, suggesting that there was no apparent increase in the expression of these genes in response to salt stress in *A. ilicifolius* under natural conditions (Additional file 6b). In contrast to terrestrial glycophytes, mangrove trees are more adaptive to moderate levels of salt than to freshwater environments [53]. Transcription analysis of *Ceriops tagal* has also revealed that most salt-induced genes are involved in long-term strategies in response to short-term high-salinity stress [54]. A total of three genes were more highly expressed in *A. ilicifolius* than in *A. leucostachyus*. Two of the three were homologous to the gene encoding gamma-hydroxybutyrate dehydrogenase (GHBDH), which functions in oxidative stress tolerance in *Arabidopsis thaliana* [55–57]. In contrast with *A. leucostachyus*, which was collected under rainforest canopies, *A. ilicifolius* was sampled from frontal thickets on the stream edges of recently accreting estuarine banks of Zhujiang River. Direct sunlight may cause the higher expression of these two oxidative stress-related genes in *A. ilicifolius*. The third gene, Ail_c52163_g1_i1, is homologous to the gene encoding chloroplast stem-loop binding protein of 41 kDa, which is involved in oxidative stress tolerance and chloroplast RNA metabolism in seedlings. In contrast, the expression of Ail_c51326_g2_i1, the product of which is CDSP32, was two-fold higher in *A. leucostachyus* compared with *A. ilicifolius*. This result is in agreement with Parida [58], who has reported that polypeptides with a molecular weight of 32 kDa are decreased in *B. parviflora* as a result of salt stress. However, in this study, only one individual was collected for each species with no biological replicates; therefore, the comparison of expression levels between the two species are not that reliable and only represent preliminary findings that may be expanded upon in a further investigation based on more samples.

## Conclusion

*Acanthus* is the only genus consisting of both mangrove and terrestrial species. In this study, we characterized the transcriptomes of one mangrove species in *Acanthus*, *A. ilicifolius*, and its terrestrial relative *A. leucostachyus*. This comparative transcriptome analysis has greatly enriched the current knowledge of the origin of the mangrove species in *Acanthus* and their adaptive evolution to abiotic stresses in the intertidal environment. Phylogenetic analysis indicated that the initial entry of the mangrove species in *Acanthus* into the intertidal environment might have occurred during the transgression in the Early to Middle Miocene. However, our study found that positive selection plays an important role in the process of adaption to harsh intertidal zones.

## Methods

### RNA extraction and transcriptome sequencing

One individual was collected under natural conditions for each of the two species, *A. ilicifolius* and *A. leucostachyus*. The sample of *A. ilicifolius* was collected near an estuary in Nansha, Guangzhou, while that of *A. leucostachyus* was sampled in from the Xishuangbanna Tropical Botanical Garden in Xishuangbanna. Both samples were of good health and no disease, and collected at the natural growth conditions without any artificial stress treatment. Fresh leaves and roots tissues from each sample were collected in the morning and pooled with approximately equal quantities for RNA extraction. Total RNA was extracted from each pooled sample using a modified CTAB method [59]. RNA samples were sequenced using an Illumina HiSeq 2000 platform.

### De novo assembly and functional annotation

After obtaining clean reads without adaptor-ligated regions by Illumina sequencing, we first filtered out reads of low quality. For each read, the longest contiguous segments with base qualities of 20 or greater and lengths of 50 bp or greater were extracted using the Dynamic Trim and LengthSort script in SolexaQA_v.2.2, respectively [60]. A *de novo* transcriptome assembly was carried out with the latest version of the Trinity program (trinityrnaseq_r20140413), with a minimum k-mer coverage of 2 [14]. To remove redundancy, two re-assembly programs, TGICL-2.1 [15] and CD-HIT [16], were employed to reassemble highly similar transcripts with identity thresholds of 0.94 and 0.90, respectively. Due to likelihood of the misassembly of contigs with low coverage and depth, we remapped the reads to the assembled transcripts using Bowtie2 [61] and SAMtools [62] and removed those contigs with less than 75 % of sites with at least 2× from the datasets. Open reading frames (ORFs) for each contig were determined by TransDecoder, a downstream software of Trinity, with the criteria that only coding regions containing at least 100 amino acids were retained for subsequent analysis. To identify the most descriptive annotations for the datasets, all of the assembled unigenes were searched against Swiss-Prot database in AgBase [17] with a cutoff e-value of 1e-6. Gene Ontology (GO) [63] classifications were performed with WEGO [64].

### Identification of orthologous contigs

The genomic data sets of *Sesamum indicum* and *Mimulus guttatus* were download from the Sinbase (http://www.ocri-genomics.org/Sinbase/index.html) and JGI databases (http://genome.jgi-psf.org/mimulus/mimulus.home.html), respectively. Putative orthologs between each pair of the three species, the two *Acanthus* species and *Av. marina*, were retrieved from the proteome sequences using OrthoMCL [65]. For each pair, we performed an all-versus-all protein sequence similarity search using BLASTP, with an e-value cutoff of 1e-10 and an identity threshold of 50 %. Similar sequences were subsequently clustered with the Markov Clustering Algorithm and grouped into species families and single-copy gene families. For each pair of orthologs, a parallel tool, ParaAT (Parallel Alignment and back-Translation), was applied to perform protein-coding DNA alignments [66] and multiple alignments of protein sequences with ClustalW2 using the default parameters [67]. All of the gaps generated during the alignment were deleted, and the amino acid alignments were back-translated to the corresponding codon sequences by PAL2NAL [68].

### Phylogenetic analysis and estimations of divergent time between different species

The synonymous substitution rate (Ks) and non-synonymous rates (Ka) of the orthologs between each pair of *A. ilicifolius, A. leucostachyus* and *Av. marina* was estimated by using KaKs_Calculator [69] with the YN00 model [70]. The non-synonymous to synonymous substitution ratio (Ka/Ks) was calculated for the orthologs between *A. ilicifolius* and *A. leucostachyus*. To infer the phylogenetic relationship of the genus *Acanthus*, we constructed phylogenetic trees for *A. ilicifolius*, *A. ebracteatus* and *A. volubilis* and three terrestrial species *A. leucostachyus*, *A. mollis* and *A. montanus*. Primers corresponding to three nuclear loci with a Ks value of approximately 0.145 and low heterozygosity that could accurately determine the phylogenetic relationship of *A. ilicifolius* and *A. leucostachyus*, were designed from the transcriptome data and screened for further polymerase chain reaction (PCR) amplification (Additional file 8). The nrITS (including ITS-1, the 5.8S rRNA gene, and ITS-2) region and three chloroplast fragments (*trn*V-*trn*M, *rpl*-*rps* and *trn*L-*trn*F) were also included and amplified with universal primers (Additional file 8). All of the sequences used in this study were deposited in GenBank with the accession numbers were [GenBank: KM652490-KM652552] and [GenBank: KM888781-KM888791] (Additional file 9). These sequences were aligned with CLUSTALX version 1.7 [71] and manually adjusted using SeqMan (version 7.10; DNAStar, London, UK). All the sequences were combined in series as one dataset. The phylogenetic trees were constructed with the combined dataset by Maximum likelihood (ML) method in PAUP* 4.0b [72]. *Av. marina* was used as an outgroup based on previous taxonomy (McDade, 1999 [73]; Schäferhoff, 2010 [74]). Before the tree construction, appropriate nucleotide substitution models were selected from the 56 models of nucleotide substitution for the combined dataset with Modeltest 3.7 (Posada, 2004 [75]). TVM + G was identified as the best-fit models for the combined dataset based on the Akaike Information Criterion. Bootstrap analyses were performed with 1000 replicates for the heuristic search with tree bisection–reconnection branch swapping and the maxtrees was set to 500. Furthermore, we calculated Kimura two-parameter distances [76] for the nrITS and chloroplast fragments, respectively, and the pairwise Ks value by Li-Wu-Luo method [77] for the three nuclear genes in Mega 5.0 [78].

Based on the 2104 pairs of orthologs among the five species *A. ilicifolius*, *A. leucostachyus*, *Av. marina*, *S. indicum* and *M. guttatus*, we estimated the divergence times between the mangrove and non-mangrove species of *Acanthus*, as well as between *Acanthus* and *Avicennia*, using the mcmctree program of PAML4.8 [18], using the independent rates model with the HKY85 substitution model. The first and second codons of each sequence were combined into two datasets, respectively. The likelihood was calculated using the pruning algorithm of Felsenstein. The program of the same parameters was run independently twice with different seeds to ensure convergence. Tripp and McDade [79] have used the record of a fossil taxon of *Avicennia* (38 MYA) to identify the most recent common ancestor (MRCA) of the extant *Avicennia* species and have estimated the divergent time of the *Avicennia* clade and Acanthaceae s.s. as 82 MYA, which is quite larger than the estimations of Bremer et al. (54 MYA) [80] and Bell et al. (41 MYA) [81]. Their approximation may have been an overestimation of the origin time of the genus *Avicennia*, leading to an overestimation of the divergence time of the *Avicennia* clade and Acanthaceae s.s.. In this study, we employed the divergence time of Scrophulariaceae (approximately 68–75 MYA) as fossil calibration information based on the results of the phylogenetic dating of Asterid Flowering Plants [80]. The appearance time of a fossil taxon *A. rugatus* Reid and Chandler (approximately 33.7–28.8 MYA) [82], the most recent common ancestor of *Acanthus*, was also considered as an indirect calibration.

### Detecting genes under positive selection in *A. ilicifolius*

The branch-site model implemented in the codeml module of PAML was used to identify candidate genes under positive selection along the branch of *A. ilicifolius* from 2245 pairs of orthologs among four species, including *A. ilicifolius*, *A. leucostachyus*, *Av. marina* and *S. indicum* [18–20]. The branch-site model A1 in combination with the BEB procedure was specified by setting the branch of *A. ilicifolius* as the “foreground branch” and all other branches in the tree as “background” branches. We compared the model A1 with and without ω fixed to 1 to determine whether the branch of *A. ilicifolius* was under positive selection. We applied the likelihood ratio test (LTR) to assess the reliability of the results. A p-value of less than 0.05 was considered significant to ensure for the power and accuracy of the LRT for detecting the signals of positive selection. In addition, Bonferroni’s multiple testing correction was applied to control the false discovery rate, with a threshold of lower than 0.05. Retained candidate genes were annotated with the GO and Kyoto Encyclopedia of Genes and Genomes (KEGG) database [21] using the automatic annotation tool Blast2GO [83] with a cutoff e-value of 1e − 6. Transcript abundances were calculated using TopHat program [84], which outputs read counts and the number of fragments per kilobase of exon per million fragments mapped (FPKM) [22], and significance of the difference between the two species was tested using the Fisher’s exact test.

## Availability of supporting data

The data sets supporting the results of this article are included within the article and additional files.

## Additional files

Additional file 1:
**Length distribution of the contigs after removing redundancy in** ***Acanthus ilicifolius*** **(red) and** ***A. leucostachyus*** **(blue).** (PDF 261 kb) Additional file 2:
**Pairwise synonymous substitution rate (Ks) of the three nuclear loci for the six** ***Acanthus*** **species.** The abbreviations were listed in Additional file 9. (XLSX 9 kb) Additional file 3:
**Pairwise Kimura two-parameter distances of nrITS for the six** ***Acanthus*** **species.** The abbreviations were listed in Additional file 9. (XLSX 9 kb) Additional file 4:
**Pairwise Kimura two-parameter distances of the combined chloroplast fragments for the six** ***Acanthus*** **species.** The abbreviations were listed in Additional file 9. (XLSX 9 kb) Additional file 5:
**List of the 13 pairs of orthologs between** ***Acanthus ilicifolius*** **and** ***A. leucostachyus*** **with Ka/Ks ratios significantly larger than 1.** – indicates that the orthologs cannot find their orthologous genes in Arabidopsis. (XLSX 10 kb) Additional file 6:
**GO distribution of the 99 candidate positively selected genes (PSGs) and FPKM of 23 stress-responsive genes in** ***Acanthus ilicifolius.*** **a** GO distribution of the 99 candidate PSGs in *Acanthus ilicifolius*. **b** FPKM of the 23 PSGs involved in salt, high temperature and UV stress tolerance and seedling germination and embryo development for *A. ilicifolius* (red) and *A. leucostachyus* (blue). Red double asterisks indicate the transcript expression in *A. ilicifolius* is higher than that in *A. leucostachyus* with p-value less than 0.01, while blue double asterisks indicate the transcript expression in *A. ilicifolius* is less than *A. leucostachyus* with p-value less than 0.01. (PDF 483 kb) Additional file 7:
**List of the 99 candidate positively selected genes (PSGs) in** ***Acanthus ilicifolius*** **.** – indicates that the gene cannot find their orthologous genes in Arabidopsis. Gene ID of the 23 PSGs involved in salt, high temperature and UV stress tolerance, seedling germination and embryo development is shown in bold. (XLSX 14 kb) Additional file 8:
**Gene IDs, sequence lengths, gene descriptions and primer sequences used in this study.** (XLSX 9 kb) Additional file 9:
**Sampling information and GenBank accession numbers of the six** ***Acanthus*** **species and the outgroup** ***Avicennia marina*** **used in this study.** The sequences of the three nuclear genes of *Av. marina* were obtained from the unpublished genomic data set of *Av. marina* and were not deposited in Genbank. (XLSX 9 kb)

## Abbreviations

BS: Bootstrap support
CI: credibility interval
FPKM: Fragments per kilobase of exon region in a given gene per million mapped fragments
GO: Gene ontology
Ka: Non-synonymous rates
Ka/Ks: Non-synonymous to synonymous substitution ratio
KEGG: Kyoto Encyclopedia of Genes and Genomes
Ks: Synonymous substitution rate
MRCA: Most recent common ancestor
MYA: Million year ago
PSGs: Positively selected genes
RSO: Reactive oxygen species
UV: Ultraviolet
