# Supplementary figures and images for: Transcriptome analysis of the Holly mangrove Acanthus ilicifolius and its terrestrial relative, Acanthus leucostachyus, provides insights into adaptation to intertidal zones
Source: BMC Genomics. 2015 Aug 14;16(1):605. doi: 10.1186/s12864-015-1813-9 (PMC4536770; doi:10.1186/s12864-015-1813-9)

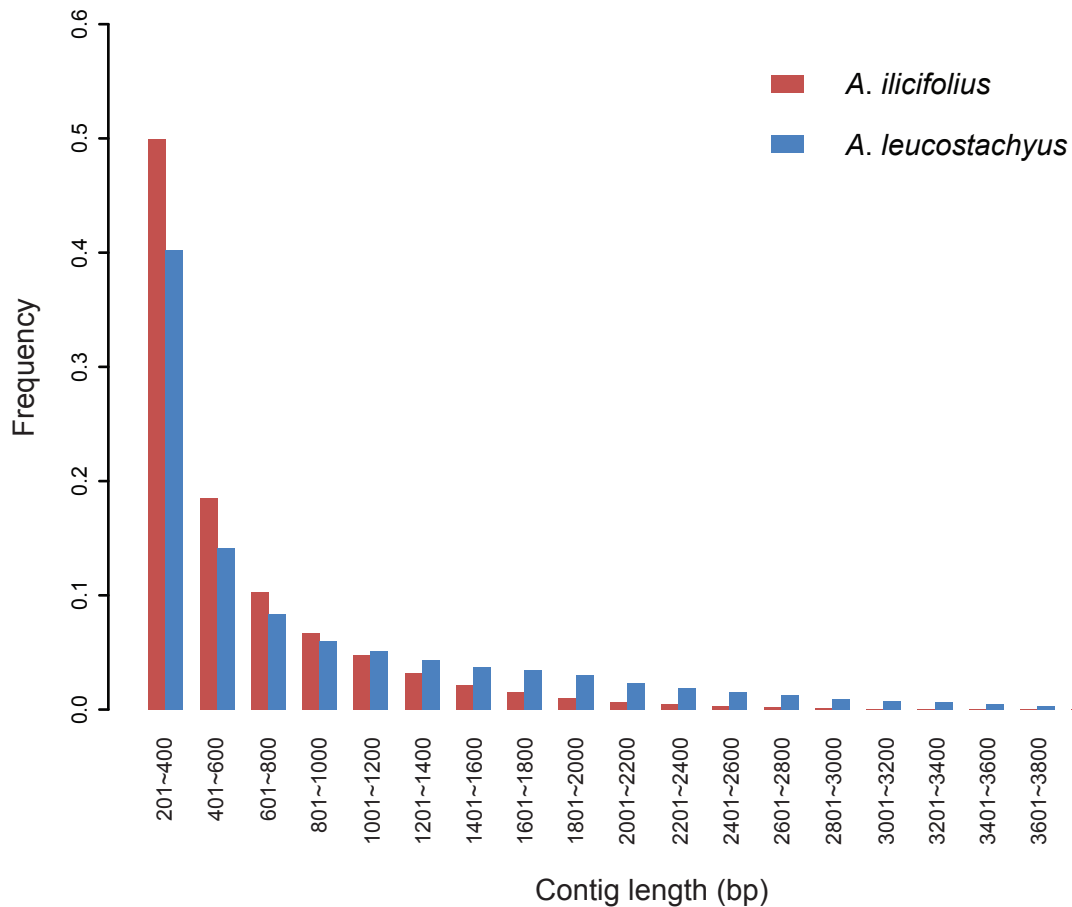

Supplement: Additional file 1: — Length distribution of the contigs after removing redundancy in Acanthus ilicifolius (red) and A. leucostachyus (blue). (PDF 261 kb) [file 12864_2015_1813_MOESM1_ESM.pdf]

**a**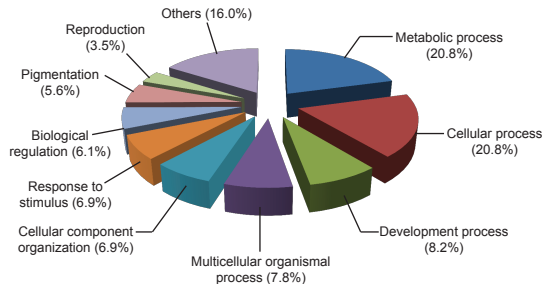**b**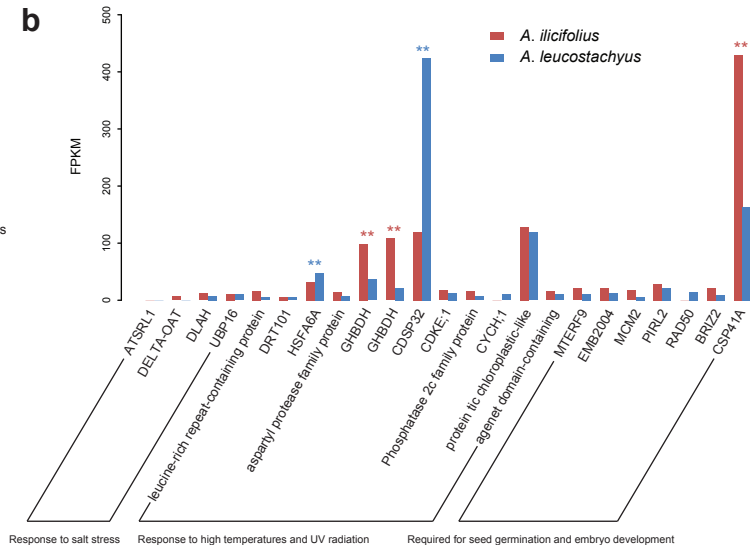

Supplement: Additional file 6: — GO distribution of the 99 candidate positively selected genes (PSGs) and FPKM of 23 stress-responsive genes in Acanthus ilicifolius. a GO distribution of the 99 candidate PSGs in Acanthus ilicifolius. b FPKM of the 23 PSGs involved in salt, high temperature and UV stress tolerance and seedling germination and embryo development for A. ilicifolius (red) and A. leucostachyus (blue). Red double asterisks indicate the transcript expression in A. ilicifolius is higher than that in A. leucostachyus with p-value less than 0.01, while blue double asterisks indicate the transcript expression in A. ilicifolius is less than A. leucostachyus with p-value less than 0.01. (PDF 483 kb) [file 12864_2015_1813_MOESM6_ESM.pdf]
